# Within-Family Environment and Cross-Fostering Stress Affect Behavior and Physiology in Wild Cavies (*Cavia aperea*)

**DOI:** 10.3389/fpsyg.2020.00178

**Published:** 2020-02-11

**Authors:** Sabine Kraus, Fritz Trillmich, Anja Guenther

**Affiliations:** ^1^Department of Animal Behaviour, Bielefeld University, Bielefeld, Germany; ^2^Max Planck Institute for Evolutionary Biology, Plön, Germany

**Keywords:** personality, early development, individual differences, family effects, cross-fostering, developmental plasticity, rank size

## Abstract

Stability of personality traits is well-documented for a wide variety of animals. However, previous results also suggest that behavioral phenotypes are plastic during early ontogeny and can be adaptively shaped to the social environment. In cavies (*Cavia aperea*), it has already been documented that the size at birth relative to siblings (size rank) greatly influences various behavioral and physiological traits that last at least until independence. The aim of the current study was (1) to investigate if behavioral and physiological differences between pups of the same litter persist until after independence and influence development long-lasting, (2) to determine the potential plasticity in response to changes in the early within-family environment by cross-fostering pups either to the same, a lower, or a higher size rank in a foster-family. We measured three behavioral traits (number of interactions with a novel object, distance moved in an open field, struggle docility) and two physiological traits (resting metabolic rate and basal cortisol levels). We predicted that cross-fostering into a litter where pups occupy the same size rank would not change the expression of traits. Cross-fostering to a different size rank should not influence the expression of traits if repeatability measures indicate low plasticity. Alternatively, if the traits are plastic, animals should adjust trait expression to fit with the size rank occupied in the foster litter. Initial differences in struggle docility, distance moved in an open field and in baseline cortisol concentration between pups of different size-ranks did not remain stable beyond independence. In addition, we found remarkable plasticity of the measured traits in response to cross-fostering to the same, a smaller or larger size-rank, suggesting that differences between pups are more the result of social constraints leading to adaptive shaping of individual phenotypes within a family. We also found a significant influence of the cross-fostering procedure itself. Cross-fostered individuals were less bold, grew slower and showed elevated resting metabolic rates. This finding suggests a cautious interpretation of previous cross-fostering studies and stresses the need for proper control groups to reliably separate the effect of cross-fostering *per se* from those induced by an experimental treatment.

## Introduction

Stability of personality traits is well-documented for a wide variety of animals ranging from spiders (Liedtke et al., 2015) to humans (Gosling, 2001; Putnam, 2011). However, it has also been documented that environmental and in particular social influences can modify the developmental trajectory of personality traits (Sachser et al., 2018; Trillmich et al., 2018). Studies revealed that the interaction between parents and offspring (quality/quantity of parental care) (e.g., Meaney, 2001) and factors like group size and composition (for example, sex and number of siblings) of a litter or a clutch affect personality traits potentially long-lasting (e.g., Benus and Henkelmann, 1998; Dimitsantos et al., 2007; Eccard and Rödel, 2011; Naguib et al., 2011).

Parental effects, i.e., non-genetic environmental effects transmitted from one or both parents to the offspring (Mousseau and Fox, 1998) have the potential to influence offspring development during the pre- and early postnatal phase. For instance, male mice (*Mus domesticus*) raised from day 4 on in a group containing males only have as adults a more active coping style (Benus and Henkelmann, 1998). Great tits (*Parus major*) from small broods show stronger stress responses than individuals from normal sized broods and individuals from female biased broods are faster explorers than those from male biased broods (Naguib et al., 2011). Such effects might arise due to constraints such as limited food supply, or may represent adaptive shaping of offspring to environmental and social conditions they are likely to encounter in the future (Gluckman et al., 2008; Nettle et al., 2013; Bateson et al., 2014).

In oviparous species, the earliest possibility of information transmission in development occurs even before fertilization. Mothers can change the composition of the egg, either by differentially allocating resources to the embryo (e.g., vitamins, nutrients) or by signals like hormones (e.g., Schwabl, 1996; Groothuis et al., 2005b; von Engelhardt and Groothuis, 2011; Groothuis and Taborsky, 2015). In mammals, there is a much greater opportunity for information exchange through the possibility for longer and reciprocal exchange of substances between mother and offspring during gestation (Del Giudice, 2012). Postnatally, parents can affect their offspring’s development by differential food provisioning (Groothuis et al., 2005a). In mammals, mother’s milk is an important pathway for nutrient transfer and hormone signaling that potentially influences offspring growth and personality (Peaker and Neville, 1991; Catalani et al., 2011; Hinde et al., 2015).

In addition to parental effects, the development of a certain behavioral and physiological type is known to be influenced by litter size and concomitant difference in competitive regime (Eccard and Rödel, 2011). Mammalian siblings might exert influences on each other and even the maternal state while still *in utero* (vom Saal, 1989). After birth, competition among siblings for limited resources is known to be an important mechanism shaping phenotypic development (Stockley and Parker, 2002). For example, differences among littermates are suggested to contribute to long-term individual differences in physiology and behavior. In rabbits (*Oryctolagus cuniculus*), individuals that occupy the periphery in the litter huddle are more proactive than their intermediate or central littermates (Reyes-Meza, 2011). Havier newborn rat pups are braver and more explorative (Rödel and Meyer, 2011). In humans, character displacement within the family is known to exert long-term effects and often carry-over until adulthood (Sulloway, 2010).

We have previously documented that relative size at birth in comparison to siblings exerts a major influence on various behavioral and physiological traits that last at least until independence in cavies (*Cavia aperea*; Guenther and Trillmich, 2015). Animals born as the largest pup in the litter were bolder, coped with stress more actively and had lower baseline blood cortisol concentrations than their siblings (Guenther and Trillmich, 2015). However, prior results also suggest that behavioral phenotypes remain plastic over a long period of time during early ontogeny and may be adaptively shaped during maturation (Guenther and Trillmich, 2013; Sachser et al., 2013; Guenther et al., 2018). Here, we raise two questions: (1) Do differences in behavioral and physiological phenotype among pups persist after independence and maturation when offspring have left the family environment? (2) Do pups express plastic responses and adapt to a change in social environment within the family, i.e., do they assume the behavioral and physiological characteristics of a novel size rank when cross-fostered?

Cross-fostering is a frequently used method to study treatment effects (e.g., Meek et al., 2001; Kessler et al., 2011), to test life history theory predictions (e.g., Rehling and Trillmich, 2007; Crino et al., 2020), to disentangle genetic from non-genetic effects (e.g., Francis et al., 2003; Groothuis et al., 2005a) or to disentangle pre- and postnatal effects (e.g., Horton, 1985; Wolf et al., 2011). Although cross-fostering experiments have proven to be an important tool to study the programing of neural, behavioral and physiological development in mammals (McCarty, 2017), recent studies show that cross-fostering itself might induce changes in the developmental trajectory (e.g., Bartolomucci et al., 2004). Therefore, the authors suggest to carefully interpret results from cross-fostering studies and include proper controls in the experimental design. Tests of effects due to cross-fostering have so far been limited to mice and rats – altricial species (Barbazanges et al., 1996; Bartolomucci et al., 2004; Hager et al., 2009; Matthews et al., 2011). There might, however, be differences between altricial and precocial species with respect to the influence of early experiences. In altricial species, the young are born after a short gestation and much of the development (neural, physiological) occurs after birth (Blumberg and Sokoloff, 1998; Sisk and Foster, 2004; Sisk and Zehr, 2005). The young require substantial maternal care, so the early postnatal period is an especially favorable time for early experiences to affect the development of young. By contrast, in precocial species, like guinea pigs (*Cavia aperea* f. *porcellus*) and their ancestors the wild cavies (*Cavia aperea*), the gestation period is long and pups are born highly developed and less dependent on milk intake than altricial young. Precocial young require less maternal care, and so the possibilities for maternal shaping of the behavior of the infant are relatively limited compared to altricial species. On the other hand, the long pregnancy offers great scope for prenatal influences on development (Rood and Weir, 1970; vom Saal, 1989; Sachser et al., 2013).

We therefore compare pups that are raised by their genetic mother, representing an undisturbed control group, and pups that are fostered to an unknown foster-family but occupy the same size rank in the foster-litter as in their natal-litter to test for effects of cross-fostering.

Given that we previously found a substantial repeatability of several traits correlated with size rank at birth, we predicted that cross-fostering into a litter where pups occupy the same size rank would not change the expression of traits. Cross-fostering into a different size rank (lower or higher than in their litter of origin) should also not influence the expression of the traits, if the repeatability measures indicate low plasticity of these traits. Alternatively, if the traits were highly plastic, animals should adjust trait expression to fit with the size rank occupied in the foster litter. As a control, we also observed a group of animals that remained in the litter of origin to test if the results of our previous study (Guenther and Trillmich, 2015) could be repeated.

## Materials and Methods

### Animals and Housing

The animals used for this study originated from a captive breeding stock of wild cavies (*Cavia aperea*) kept and bred in Bielefeld since 1981. Wild-caught animals are crossed into the population every few generations to prevent potential effects of inbreeding or domestication. For breeding, females were transferred from outdoor enclosures under natural photoperiod and temperature to climate chambers located indoors. Females were housed singly in 0.8 m^2^ enclosures equipped with a shelter, a rough stone, a feeding dispenser and a water bottle. Water, fresh hay and guinea pig pellets (Höveler, Germany) were available *ad libitum*. In addition, vitamin C (1 g/l) was added to the drinking water once a week and animals were supplemented with fresh greens such as carrots, bell pepper or apples, four times a week. Rooms were kept at 20 ± 2°C throughout the experiment. Initially, the photoperiod was set to 12:12 light:dark (L:D) for 4 weeks to reset information about the photoperiod in females. Thereafter, a male was introduced for 2 weeks and the light:dark cycle was set to 9.5 L:14.5 D. 15 min of light were added every 9 days to simulate the spring photoperiod. This was done because photoperiod is known to influence offspring development regarding life history, physiology, and behavior in cavies (Guenther et al., 2014; Rübensam et al., 2015; Finkemeier et al., 2016) and our experiments were run at different times of the year. Since pregnancy of cavies lasts for 60 days, offspring were born under 11:45 to 12 h light and experienced increasing photoperiod until the end of the experiments.

58 days after introducing males for breeding, we started to check enclosures 6 days a week for newborn pups. All pups were initially given a haircut for individual recognition. After weaning (24–30 days of age), pups were marked permanently with a subcutaneous pit tag (ID 100, TROVAN, passive transponder system, Euro ID, Weilerswist, Germany).

We conducted two experiments. *Experiment I* was run to test for reproducibility of size rank differences in non-cross-fostered pups as found in an earlier study (Guenther and Trillmich, 2015). Here, 22 females were bred, 19 of which gave birth (Table 1). In total, 45 offspring were tested for behavioral and physiological development. In *Experiment II*, 48 females were bred, of which 44 gave birth (Table 1). In this experiment, pups were cross-fostered shortly after birth (see experimental procedure). The aims of this experiment were (a) to test if cross-fostering to a higher or lower size rank would influence the early behavioral and physiological development long-lasting, i.e., if juveniles would adjust their phenotype to their new social niche, and (b) to test, if predictable size rank differences remain stable after cross-fostering, i.e., to control for any potential effects of the cross-fostering procedure on phenotypic development.

**TABLE 1:** Overview of the number of females used for breeding (N females breeding), the number of females that gave birth (N litters produced) and number of pups in brackets.

	**N females breeding**	**N litters produced (# of pups)**	**N litters entering experiment**	**N pups entering experiment (male/female)**	**N pups cross-fostered**
Experiment I	22	19 (50)	18	45 (23/22)	–
Experiment II	48	44 (89)	34	68 (34/34)	Same: 29 Up: 19 Down: 20

*Litters were excluded, if they consisted of a single pup only, if two or more pups died before weaning or if there were no size- and age-dependent matches for cross-fostering of pups (*Experiment II*). Pups cross-fostered: “up” means cross-fostered into a litter so that its size rank was higher than in the birth litter; “same” means fostered to another litter but to the same size position as in the original litter; “down” cross-fostered so that it occupied a lower size rank in the new litter than in the original litter.*

### Experimental Procedures and Timeline

Pups were assigned a size rank in their litter of birth based on birth mass. Bigger pups are located closer to the cervix *in utero* and hence are also born before their smaller siblings (Schumann et al., 2014). In *Experiment II*, pups were cross-fostered within 3 days after birth depending on the availability of same-aged litters. Pups were distributed to new litters so that each foster-family consisted only of unfamiliar pups, i.e., all pups originated from different litters to ensure that all animals had equal starting conditions. Three days after pups had been introduced into their foster families, they were weighed again to estimate the effect of cross-fostering on body mass development. In *Experiment I*, pups were weighed a second time at 4 days of age.

A first round of behavioral and physiological testing was conducted around the time of weaning (i.e., at an age of 19–30 days) when pups were still kept together with their foster-family (*Experiment II)* or their family of birth (*Experiment I*). In total, each pup was tested in three behavioral (Open Field, Novel Object, Struggle) and two physiological tests (resting metabolic rate – RMR, baseline blood plasma cortisol concentration – CORT). Tests were conducted in random order and each pup was tested in only one test per day. After each test, the animal was given a minimum of 24 h rest to prevent any carry-over effects between tests. Animals completed all tests within 10–12 days. Behavioral tests were conducted between 9–12 am or 2–5 pm similar to previous studies where no time-of-day effect was found (Guenther and Trillmich, 2013; Guenther et al., 2014). CORT was taken at noon ± 10 min and RMR was measured between 9 am and 6:30 pm.

After the first test round had been completed for all pups in the litter, this litter was separated from its (foster) mother (at age 24–30 days). Pups were weighed to determine daily growth rate until weaning. Pups were thereafter kept in groups of two together with an unfamiliar and unrelated same-sex pup in identical enclosures until the end of the experiment.

Shortly after sexual maturation (∼50 days Guenther and Trillmich, 2013; Guenther et al., 2014), a second round of behavioral and physiological testing was conducted similar to the first round. Tests were conducted between 55 and 75 days of age for all animals to test for long-term effects of the early social niche. Males of this species often become aggressive when reaching sexual maturity. When this happened, we separated male pairs using wire-mesh so that animals still had visual and olfactory contact with each other but were prevented from interacting physically.

### Physiology

#### CORT

Blood samples (∼70 μl) were taken within 3 min after capturing the animal to avoid a rise of baseline concentrations due to handling stress (Romero and Reed, 2005). One experimenter held the animal on its lap while a second experimenter collected blood from the marginal ear vein into heparinised capillaries. Only one animal per enclosure was tested per day since capturing may stress the co-housed animals. Blood was centrifuged for 5 min at 10000 rpm and then stored at −20°C until further analyses.

Analysis was performed using a competitive enzyme immunoassay (RE52061 IBL, IBL International GmbH, Hamburg, Germany) using specific antibodies against cortisol (for further details see Kaiser et al., 2003). The antibody that we used cross-reacted with relevant steroids as follows: Prednisolone 29.8%, 11-desoxycortisol 8.48%, cortisone 4.49%, prednisone 2.12%, corticosterone 1.99%, 6b-hydroxycortisol 1.03%. Samples were evenly distributed across seven assays. The intra-assay% CV was 4.2% and the inter-assay% CV was 6.7%.

#### RMR

Two animals could be measured at the same time to assess resting metabolic rate. Each animal was placed into a metabolic chamber (transparent Plexiglas, 18 cm × 28.5 cm × 18 cm) located in a climatized cabinet (Rubarth Apparate, Laatzen, Germany). Measurements lasted for 3.5 h and were conducted under low light conditions and at 20 ± 1°C at the lower end of the thermoneutral zone. We used open flow respirometry with a continuous air flow of outside air of about 80 l/h (Mass Flow Meter FM 360, Tylan, Corp., Torrance, CA, United States). Oxygen consumption and CO_2_ production were measured. Outside air was pumped through metabolic chambers under ambient pressure and thereafter continued into two successive coolers (M & C Cooler, Ratingen, Germany) for drying. Additional drying was achieved using scrubbers (Drierite, Fluka, Steinheim, Germany). For the measurement of O_2_ and CO_2_, a subsample of air flowed at 600 ml/min through an O_2_ analyzer (Oxzilla FC, Sable Systems, Henderson, NV, United States) and a CO_2_ analyzer respectively (Maihak AG, Hamburg, Germany). Chambers were measured alternately eight times for 10 min each per measurement. Between measurements of different chambers, we allowed 1 min to ensure that no air from the previous chamber was left and measured in the system. As resting metabolic rate, we used the 3-min period with the lowest stable O_2_ consumption after an initial period of 30 min, which is the time animals usually need to calm down.

### Behavior

#### Novel Object

Boldness was measured as number of interactions with an unknown object in the home enclosure. All other animals were gently removed from the home enclosure before testing. Then, a novel object was introduced approximately 20 cm from the shelter. The novel objects used for testing were a green egg cup in the first and a yellow rubber duck in the second test round. Interactions of the test animal with the object were video-recorded for 1 h.

#### Open Field

Fearlessness was measured as the distance moved (cm) when individuals were introduced into an open, unknown arena for 20 min. For the first 10 min, a semi-transparent shelter was present in the arena under which animals could hide. For the second 10 min, this shelter was removed from the arena. The arena was located in a silent room without any other animals present. The experimenter left the room at the beginning of the test.

#### Struggle Docility

To measure docility, an animal was gently captured and turned on its back in the hand of the observer for 30 s. We scored the time an animal actively struggled to escape this situation as a measure of stress-coping (Bonnot et al., 2018).

### Ethics Statement

All experimental procedures were in accordance with German animal protection laws. Facilities were approved (2014) by the local government authority responsible for health, veterinary and food monitoring (Gesundheits-, Veterinär- und Lebensmittelüberwachungsamt Bielefeld). The experiments were performed under license 84-02.05.20.12.246 LANUV, Germany.

### Statistical Analyses

For statistical analysis and graphing, R 3.2.3 and R 3.5.1 were used (R Development Core Team, 2008) with the package lme4 (Bates et al., 2015) for mixed models. Additionally, we used the packages ggplot2 (Wickham, 2009), effects (Fox, 2003; Fox and Weisberg, 2018), and emmeans (Lenth, 2019) to create the graphs. Residuals of the models were checked visually for distribution and variance homogeneity using Q–Q plots.

Separate models were run to analyze the first round of testing at weaning and the second round around sexual maturation. The only exceptions were the growth rates, because we had only one measurement. Furthermore, in all models of RMR, body mass at day 24 was included as additional fixed effect.

In order to calculate the effects of size rank on behavioral and physiological development in non-fostered litters, we used linear mixed models with a Gaussian distribution. Size rank (three level factor) and sex were fitted as fixed effects. Mother ID was included as a random effect, allowing random intercepts but not random slopes.

We employed linear mixed models with a Gaussian distribution in order to estimate the effect of cross-fostering to a similar size rank by including the size rank before and after cross-fostering in addition to sex. Mother ID and stepmother ID were included as random effects.

To analyze the effects of cross-fostering to the same, a higher or a lower size rank, linear mixed models contained the direction of fostering (three level factor: “same,” “up,” “down”) and sex as fixed effects. Mother ID and stepmother ID were included as random effects and a Gaussian error distribution was used.

To test for temporal consistency, we estimated repeatability for all traits by using the R-package rptR (Stoffel et al., 2017). The same model structures as described before were used to estimate adjusted repeatabilities with 1000 bootstraps for estimating confidence intervals. As we wanted to assess individual consistency, we used individual identities as grouping factor in the model. Therefore, individual ID nested within mother ID were included as random effects. We used a likelihood ratio test (LRT) for significance testing of repeatabilities.

Finally, to assess the effect of the cross-fostering procedure, we log transformed the data for RMR and CORT and used a square root transformation for the data derived from the open field test to resemble a Gaussian distribution. While growth rate resembled a Gaussian distribution, data derived from the struggle docility test and the novel object test resembled a Poisson distribution. By combining the two control groups into one dataset, the distribution of the data changed. They became more left-skewed because many individuals from the non-fostered group did not struggle and had no interactions with the novel object at weaning. In addition, we found outliers in the data for the novel object test. Therefore, we compared model results from the full dataset and a dataset in which outliers were removed. The results with or without outliers were consistent with only one exception: the difference between first and second rank individuals became significant if we excluded the outliers. Diagnostic plots revealed a better fit of the model without outliers. To assess the effect of the cross-fostering procedure we employed linear mixed models with size rank of origin (three level factor), sex, treatment (two level factor: “foster,” “non-foster”) and time of testing (two level factor: “weaning,” “maturation”) as fixed effects, as well as the two-way interaction of treatment and time of testing. Mother ID was included as a random effect.

## Results

### Effects of Size-Rank in Litter on Behavioral and Physiological Development in Natural Litters

First, we tested, if behavioral and physiological differences of the size rank within litter are reproducible (with respect to our earlier experiment, Guenther and Trillmich, 2015) with an independent set of animals and if such differences in size rank persist after maturation (*Experiment I)*.

**FIGURE 1:**
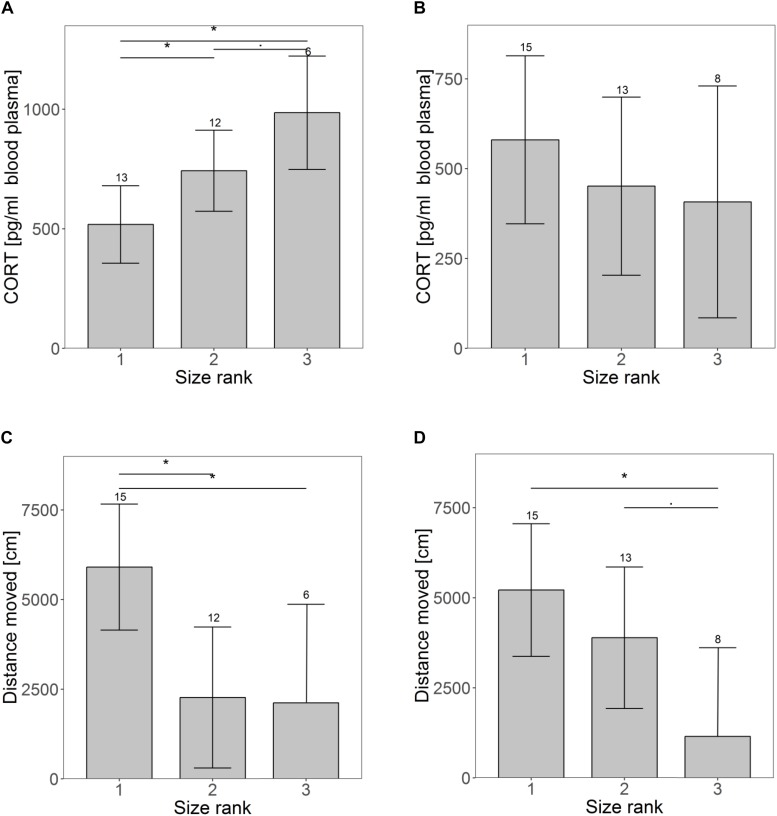
Differences in personality and physiological traits over time for pups of different size ranks (1 indicates the largest pup). Panel A and B show differences in basal cortisol levels at the time of weaning **(A)** and maturation **(B**). The images shows the distance moved in an open field at weaning **(C)** and maturation **(D)**. Shown are the estimated values derived from the mixed models on the behavioral traits ± confidence intervals (CI). Asterisks indicate significant differences among size ranks. Samples sizes are given above the CI.

Size rank did not affect growth until weaning (Supplementary Figure S1) or RMR at weaning (Supplementary Figure S2A), but males grew on average half a gram more per day compared to females (males: 5.1 g; females: 4.6 g per day; *t* = 2.07, *p* = 0.05). CORT was significantly higher for animals occupying a lower size rank in the litter at weaning (rank 1 vs. rank 2: *t* = −2.16, *p* = 0.04; rank 1 vs. rank 3: *t* = −3.51, *p* = 0.002; rank 2 vs. rank 3: *t* = −1.84, *p* = 0.08) (Figure 1A). Animals of size rank two tended to interact less with a novel object (*t* = −1.93, *p* = 0.07), showing on average only half as many interactions as animals of size rank one (largest pup) (rank 1: 7.7 ± 2.4; rank 2: 3.2 ± 2.3 interactions, Supplementary Figure S3A). Significant size rank differences were found for the distance traveled in an open field (rank 1 vs. rank 2: *t* = 3.11, *p* = 0.007; rank 1 vs. rank 3: *t* = 2.51, *p* = 0.02; rank 2 vs. rank 3: *t* = 0.10, *p* = 0.92) (Figure 1C). Smaller siblings tended to struggle more than larger ones (*t* = 1.91, *p* = 0.07, Supplementary Figure S4A). Animals of size rank two struggled on average 67% and animals of size rank three 29% more than their siblings of size rank one. Except for growth rate (higher in males), no sex effects were found at weaning.

At maturation, when animals had been together with unfamiliar and unrelated same-sex individuals for about a month, effects of the size rank were reduced. The only remaining significant effect occurred in the distance traveled in an open field (rank 1 vs. rank 2: *t* = 1.17, *p* = 0.26; rank 1 vs. rank 3: *t* = 2.95, *p* = 0.008; rank 2 vs. rank 3: *t* = 2.01, *p* = 0.058) (Figure 1D). Neither CORT (Figure 1B), nor any other traits indicated earlier size rank differences (Supplementary Figures S2B, S3B, S4B). Sex differences were only found for CORT, with males having lower CORT concentrations than females (m: 294 ± 102 pg/ml, f: 646 ± 115 pg/ml, *t* = −2.3, *p* = 0.03).

To test for temporal consistency, we estimated repeatability for all traits (Supplementary Table S1). All traits showed high temporal flexibility as none of the traits was significantly repeatable.

### Effects of Cross-Fostering and Size Rank in Litter After Cross-Fostering

We tested, if animals showed size rank differences when cross-fostered to same size ranks as in their natal litter. Comparable to non-cross-fostered animals, there were no size rank differences for growth rate, RMR, CORT or number of touches in the novel object test (Table 2). However, cross-fostering diminished the previously found size rank differences in distance traveled in open field and, opposite to the control group, we found a trend for smaller siblings to struggle less (*t* = −1.96, *p* = 0.06). At maturation, no effects of size rank were found for any trait. In addition, males and females only differed in RMR but no other trait, with males having on average a 58% higher RMR than females (m: 59.7 ± 7.54 kJ/kg^∗^day^–1^; f: 34.9 ± 6.33 kJ/kg^∗^day^–1^; *t* = 2.4, *p* = 0.03). Similar to the control animals, no trait showed a significant repeatability (Supplementary Table S1).

**TABLE 2:** Mean estimates with their corresponding standard error of behavioral and physiological traits after cross-fostering.

**Trait**	**Cross-fostered same**		**Cross-fostered up**		**Cross-fostered down**	
	**Weaning**	**Maturation**	**Weaning**	**Maturation**	**Weaning**	**Maturation**
Growth rate [g/day]	4.50.2	–	4.50.2	–	4.40.2	–
RMR [KJ/kg * day^–1^]	22.41.3	47.43.7	25.81.6	42.24.1	24.01.5	49.24.0
CORT [ng/ml]	62771.5	45568.1	76186.3	45480.9	59783.8	48578.5
# of touches	6.61.0	6.01.2	4.51.2	4.91.4	6.41.2	4.91.4
Distance moved [cm]	75261189	70091270	71341436	68071526	64641397	72481457
Struggle docility [s]	6.91.1	5.81.0	7.71.4	2.71.2	9.11.4	4.21.2

*Shown are the three different foster groups: individuals that were fostered to the same size rank than in their litter of birth and individuals that were either fostered to a higher (up) or lower (down) size rank in litter. Weaning and maturation mark the two time points at which experiments were conducted.*

### Effects of Cross-Fostering *per se*

**FIGURE 2:**
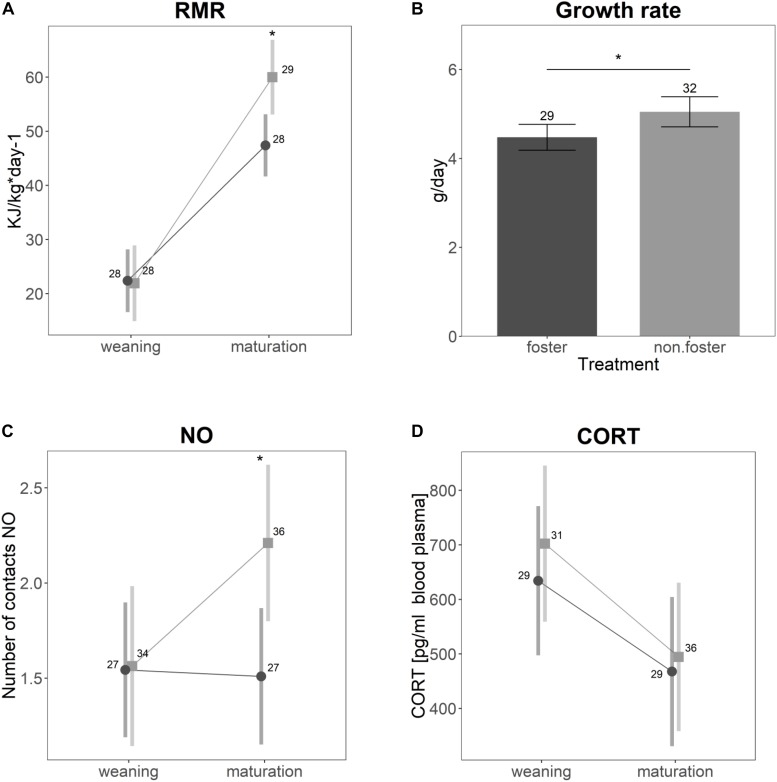
Differences between the animals of *Experiment I* (non-fostered) in light gray and animals cross-fostered to the same size ranks (*Experiment II*) in dark gray for **(A)** specific resting metabolic rate, **(B)** growth rate, **(C)** number of contacts to a novel object, and **(D)** basal cortisol levels. Shown are the estimated values derived from the mixed models ± confidence intervals. Asterisks indicate significant differences. Samples sizes are given in the plot.

Significant differences between the animals of *Experiment I* (control) and animals cross-fostered to the same size ranks (*Experiment II*) were found for growth rate and RMR (Figures 2A,B). Cross-fostered animals had lower growth rates (*t* = −2.14, *p* = 0.04) and elevated RMR (*t* = −3.7, *p* = 0.001). RMR generally increased with age (*t* = 9.4, *p* < 0.001). For cross-fostered animals, however, the increase was lower compared to non-cross-fostered animals (*t* = −3.3, *p* = 0.001). Neither CORT (Figure 2D, *t* = −0.04, *p* = 0.96), nor the distance traveled in open field (*t* = −1.3, *p* = 0.19), or struggle docility (*z* = −0.38, *p* = 0.71) differed between control and cross-fostered animals. Cross-fostered animals were less bold compared to control animals, on average touching a novel object only half as often as control animals (Figure 2C, *z* = −2.8, *p* = 0.005). In addition, the change of the number of touches between juveniles and mature animals was less strong in cross-fostered animals.

### Effects of Cross-Fostering to a Different Size-Rank in Litter

**FIGURE 3:**
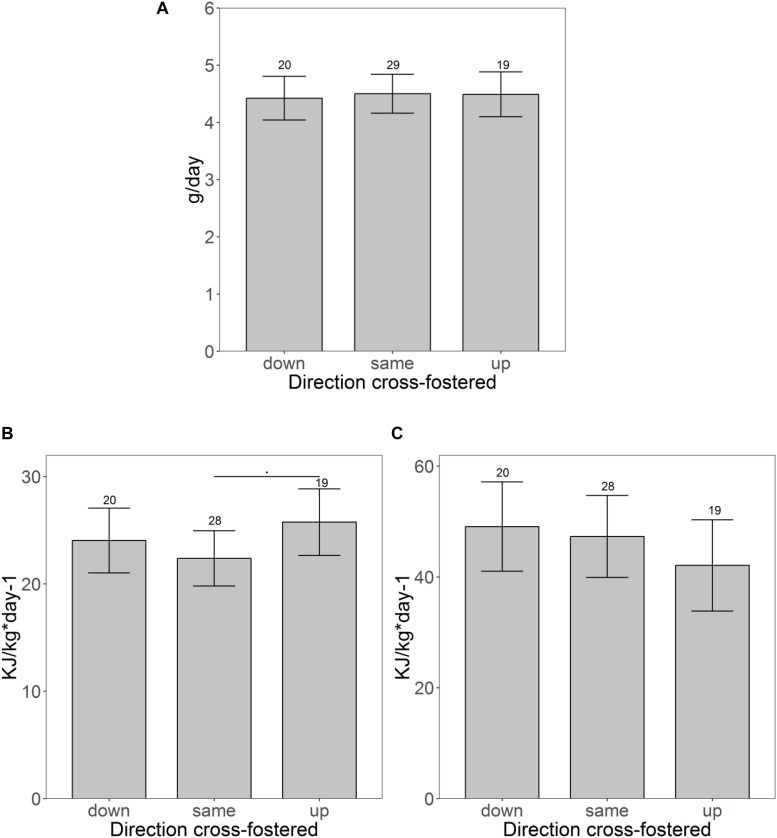
Pups fostered to different size ranks. **(A)** Shows the growth rates and **(B,C)** show differences of the specific resting metabolic rate before **(B)** and after maturation **(C)** of pups fostered to different size ranks. Shown are the estimated values derived from the mixed models on traits ± confidence intervals. Samples sizes are given above the CI.

Cross-fostering to a lower or higher size rank had only little influence on trait expression (Table 2). Neither weight development during the initial 3 days after cross-fostering (same: + 0.77 ± 0.75 g; down: + 1.84 ± 0.87 g; up: 1.04 ± 0.89 g), nor growth rate until weaning differed between pups cross-fostered to the same, a lower or a higher size rank (Figure 3A). CORT also showed no difference between pups cross-fostered up or down (same: 595 ± 85 ng/ml; up: 729.5 ± 97.5 ng/ml; down: 565.1 ± 96.6 ng/ml). RMR however, tended to be elevated in pups that were cross-fostered to a higher size rank (same: 22.5 ± 1.6 kJ/kg^∗^day^–1^; up: 25.7 ± 1.9 kJ/kg^∗^day^–1^, *t* = 7.8, *p* = 0.08). This initial trend disappeared at maturation (*t* = −1.4, *p* = 0.17) (Figures 3B,C). Males had higher RMR than females, both, at weaning, and maturation (weaning: *t* = 5.7, *p* < 0.001, maturation: *t* = 3.0, *p* = 0.004). Neither the number of touches, nor the distance moved showed any differences between pups cross-fostered to the same or to other size ranks. For struggle docility, we also found no differences at weaning but pups that were cross-fostered to a higher size rank struggled significantly less than pups cross-fostered to a same size rank at maturation (same: 5.9 ± 1.2; up: 2.8 ± 1.5, *t* = −2.0, *p* = 0.048).

## Discussion

We aimed to investigate if behavioral and physiological differences between different-sized pups of the same litter persist until after independence and influence personality development long-lasting. Furthermore, we investigated the potential for plastic responses to changes in the early within-family environment by cross-fostering pups either to a same (i.e., remain in the same size rank), a lower (i.e., becoming the smallest pup), or a higher (i.e., becoming the largest pup) position in a foster-family. We found little evidence for long-term effects but remarkable plasticity in response to changes in the social environment. Moreover, we found a significant influence of the cross-fostering procedure itself on behavioral and physiological development (as further discussed below), something that has not been investigated in much detail despite the ubiquity of this procedure in the literature.

### Plastic Responses to the Early Social Environment

As a first step, we verified that behavioral and physiological differences of the size rank within litter found in our previous study (Guenther and Trillmich, 2015) are reproducible with an independent set of animals. Pups born as the heaviest in the litter were the most fearless, bold and explorative and had the lowest plasma CORT levels around weaning. Although the effects we find in our sample were not all statistically significant (possibly due to a slightly lower sample size compared to the previous study by Guenther and Trillmich, 2015), they all point in the same direction as previously found. Comparable effects were shown in laboratory rats (*Rattus norvegicus*), where heavier pups were bolder and more explorative around weaning (Rödel and Meyer, 2011).

One possible explanation for differences in physiology and behavior in pups of different size ranks is that those may be a result of differential maternal provisioning. Studies showed that individuals receive different prenatal provisioning depending on their position *in utero* resulting in size differences between pups even several weeks before birth (Turner and Trudinger, 2000; Schumann et al., 2014). Previous findings implied a strong influence of prenatal maternal effects on personality differences of pups at an early age of 3 days (Guenther and Trillmich, 2015). Furthermore, prenatal maternal effects in guinea pigs and cavies have been shown to influence offspring behavior and physiology until adulthood (Sachser and Kaiser, 1996; Kaiser and Sachser, 1998, 2001). In domesticated guinea pigs, an unstable social environment during pregnancy causes masculinization of females and feminization of male offspring (Sachser and Kaiser, 1996; Kaiser and Sachser, 1998, 2001, 2005, 2009). Another example includes the adaptive programing to the season of birth. Animals born into autumn are less explorative, shyer and mature later, whereas animals born into spring conditions are more explorative, bolder and mature earlier (Guenther et al., 2014). As mentioned earlier (see section “Introduction”), in precocial species, the gestation period is relatively long, offering great scope for prenatal influences on development (Rood and Weir, 1970; vom Saal, 1989; Sachser et al., 2018). Mothers could adaptively program their offspring and follow a bet-hedging strategy. By diversifying the pups of a litter with different physiological and behavioral types, mothers could ensure that at least one of her offspring matches the future environmental conditions (Reddon, 2012).

Against our interpretation of an early long-term stable shaping of the phenotype, we find that these initial differences of the size rank disappear after maturation. Furthermore, none of the traits showed repeatability from the juvenile to the adolescent stage, suggesting high plasticity. This is in contrast with previous studies which demonstrated temporal consistency of the traits measured in the current study. However, in these previous studies, the phenotyping was conducted after juveniles had been separated from their mother and siblings, while in this study, the first round of testing was conducted when juveniles still lived in the family environment (Guenther and Trillmich, 2013; Guenther et al., 2014). This discrepancy suggests that differences between pups of different size within a litter largely represent the results of a size-related constraint arising from the competitive situation within the litter (Sulloway, 2010). If so, early behavioral differences among the pups may be achieved by adaptive shaping of individual phenotypes within the family. Rather than shaping an animal’s phenotype long-term, however, these differences apparently only persist as long as the social context (i.e., the family) does not change.

When being cross-fostered, juveniles neither expressed a phenotype corresponding to their natal size rank, nor to their new size rank in the litter after cross-fostering. The fact that we find remarkably high plasticity of the offspring’s phenotype implies that we only have a weak influence of prenatal maternal effects and that those effects are abolished postnatally by cross-fostering stress in the environment of a foster-mother and foster-siblings. Postnatal experiences and developmental plasticity, even later in life, offer an opportunity to readjust to the current environmental conditions. This might be necessary because informations provided by the mother earlier are not veridical, or the environmental conditions have indeed changed unpredictably, or because the offspring has emigrated to a new environment that is different from the previous one (reviewed in Sachser et al., 2011, 2013).

### Effects of Cross-Fostering

We predicted that cross-fostering into a litter where pups occupy the same size rank would not change the expression of traits. Against our expectations, however, we did not find any size rank differences anymore, indicating that the process of fostering had a great impact on the behavioral and physiological development. A cross-fostering experiment in laboratory mice showed no difference in basal plasma CORT levels but found effects of cross-fostering on behavioral and physiological parameters, particularly in males. Cross-fostered males showed an increased exploration and smaller preputial glands – testosterone-dependent organs (Bartolomucci et al., 2004). In line with that, the lower RMR together with the lower growth rate and the more reactive behavior in the novel object test for the cross-fostered animals in this study indicate that cross-fostering is stressful and influences study results to a great extent.

As mentioned before (see section “Introduction”), cross-fostering is a frequently used method and mostly used in altricial species (Barbazanges et al., 1996; Bartolomucci et al., 2004; Hager et al., 2009; Matthews et al., 2011). However, only few studies have included appropriate controls to assess whether the process of fostering itself has an effect and reported the effects of fostering *per se* on various phenotypic measures in offspring (for review see, McCarty, 2017). Matthews et al. (2011) found that cross-fostering led to profound effects on cardiovascular and metabolic function in lab mice. Fostered mice showed increased appetite, body weight, abdominal fatness (in males only) and enhanced glucose tolerance. Furthermore, fostered male mice showed an increase in systolic blood pressure compared to mice reared by their genetic mother. Moreover, a study using a QTL approach showed that phenotypic plasticity does not only originate from additive genetic dominance effects but also from epigenetic effects such as genomic imprinting (Hager et al., 2009). The authors suggested that epigenetic effects of a locus on bodyweight and growth may vary as a result of changes particularly in the maternal environment through cross-fostering. Accordingly, our results also show differences in growth rates of fostered vs. non-fostered individuals. Overall, these results show that cross-fostering stress can have very different effects on different species and even strains (e.g., Barbazanges et al., 1996; Meek et al., 2001).

Postnatal manipulations, such as cross-fostering, at different times are shown to induce different effects on behavioral or endocrine traits (Barbazanges et al., 1996). The fact that different cross-fostering protocols are applied in different studies makes it difficult to compare the effects of cross-fostering between studies. Some studies use an all-litter foster design (e.g., Francis et al., 2003), others a one pup-foster design (e.g., McCarty and Lee, 1996) or a split-foster design (e.g., van Oers et al., 2015). Moreover, the choice of control groups varies in different studies. Some studies used in-fostered groups vs. cross-fostered groups, i.e., fostering pups to the same species/strain or to a different species/strain (e.g., Gomez-Serrano et al., 2001) while others compared fostered against non-fostered groups (e.g., Meek et al., 2001) or used a combination of both approaches (Cierpial et al., 1989). To our knowledge there is no published study investigating cross-fostering effects in non-altricial species.

In the current study, we show that cross-fostering effects also occur in a precocial species with similar effects to those found in altricial species. These findings have implications for both the interpretation of previous cross-fostering studies and the design of future studies using a cross-fostering approach in precocial species. We therefore stress the importance of well-designed control groups to reliably separate the effect of cross-fostering *per se* and other correlated experimental influences from the effects a specific experiment aims to measure.

## Data Availability Statement

The datasets generated for this study are available at https://doi.org/10.4119/unibi/2940638.

## Ethics Statement

The animal study was reviewed and approved by Gesundheits-, Veterinär- und Lebensmittelüberwachungsamt Bielefeld.

## Author Contributions

AG and FT designed the experiment. AG collected the data. SK analyzed the data. SK and AG wrote the manuscript. All authors provided improvements to the manuscript and approved the final draft.

## Conflict of Interest

The authors declare that the research was conducted in the absence of any commercial or financial relationships that could be construed as a potential conflict of interest.
