# Supplementary material for: Within-Family Environment and Cross-Fostering Stress Affect Behavior and Physiology in Wild Cavies (Cavia aperea)
Source: Front Psychol. 2020 Feb 11;11:178. doi: 10.3389/fpsyg.2020.00178 (PMC7026460; doi:10.3389/fpsyg.2020.00178)
Supplement: Supplementary file 1 [file Table_1.DOCX]

**Supplementary material**

**Table S1:** Repeatability estimates (R) in non-cross-fostered (control) and animals that were cross-fostered to a similar size rank as occupied in the litter of birth. Shown are the estimates with their corresponding confidence intervals (CI) for the specific resting metabolic rate (RMR), plasma cortisol levels (CORT), number of touches the individual had with an unknown novel object, the distance moves in an open field and the time the individual actively struggle in the hand within 30 sec. Borderline significance is shown in italic.

| **Trait** | **R (CI) non-cross-fostered** | **R (CI) cross-fostered** |
| --- | --- | --- |
| RMR [KJ/d*kg^-1^] | 0 (0-0); p = 0.5 | 0 (0-0.1); p = 1 |
| CORT [ng/ ml plasma] | 0.09 (0-0.43); p = 0.33 | 0 (0-0.39); p = 1 |
| # touches novel object | 0 (0-0.36); p = 1 | 0.27 (0-0.57); p = 0.23 |
| distance moved in open field [cm] | 0.22 (0-0.56); *p = 0.05* | 0 (0-0.5); p = 1 |
| struggle docility [s] | 0.16 (0-0.49); p = 0.09 | 0.001 (0-0.45); p = 0.5 |


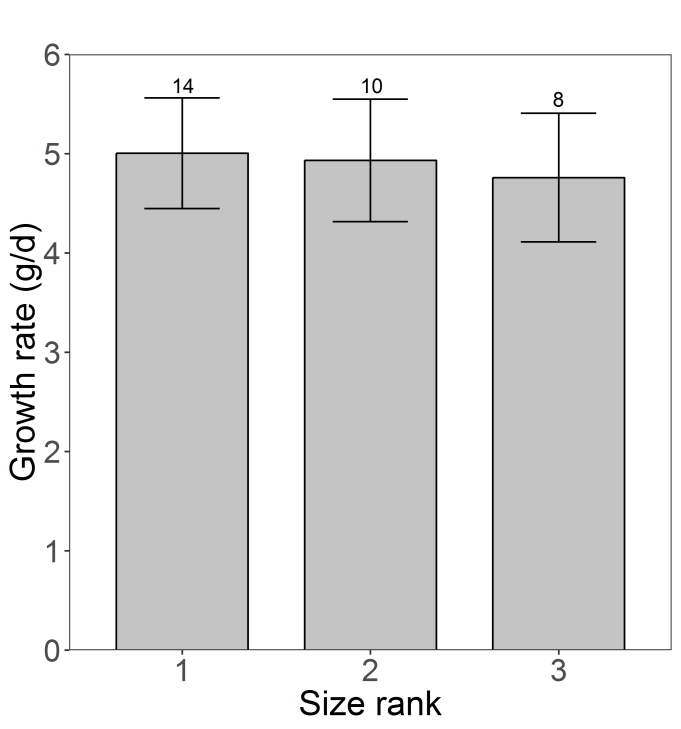


**Figure S1:** Differences in growth rate for pups of different size ranks (1 indicates the largest pup) over time. Shown are the estimated values derived from the mixed models on the behavioural traits ± confidence intervals (CI). Samples sizes are given above the CI.

**B**

**A**


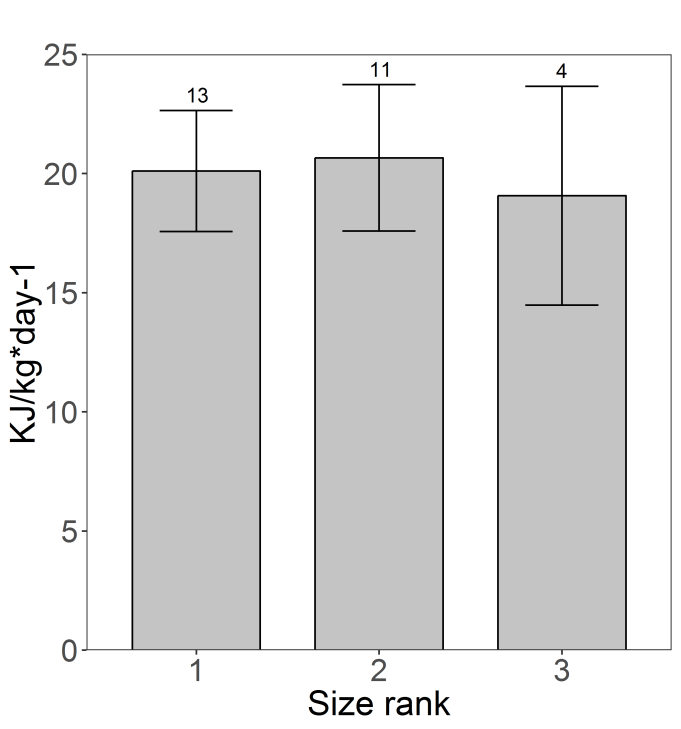

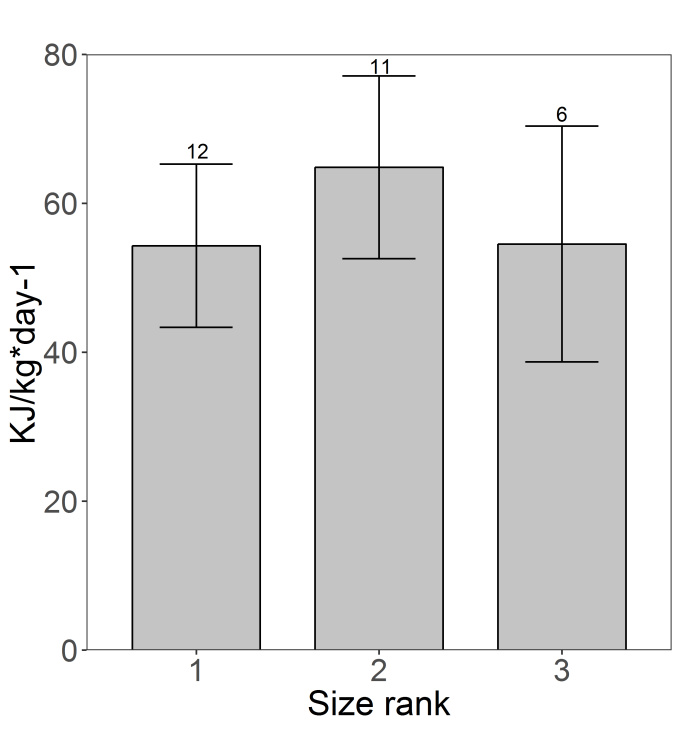


**Figure S2:** Differences in specific resting metabolic rate for pups of different size ranks (1 indicates the largest pup) over time. Panel A shows differences at the time of weaning and panel B at the time of maturation. Shown are the estimated values derived from the mixed models on the behavioural traits ± confidence intervals (CI). Samples sizes are given above the CI.


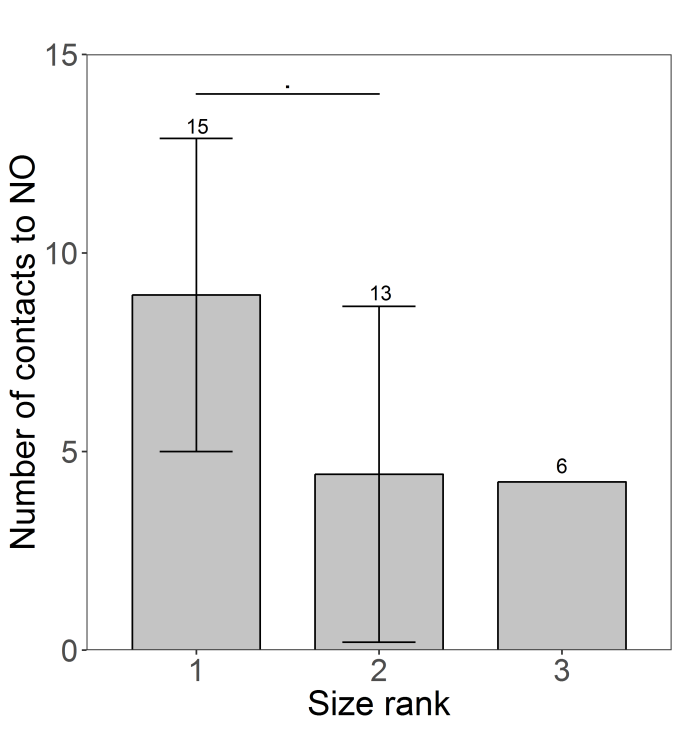

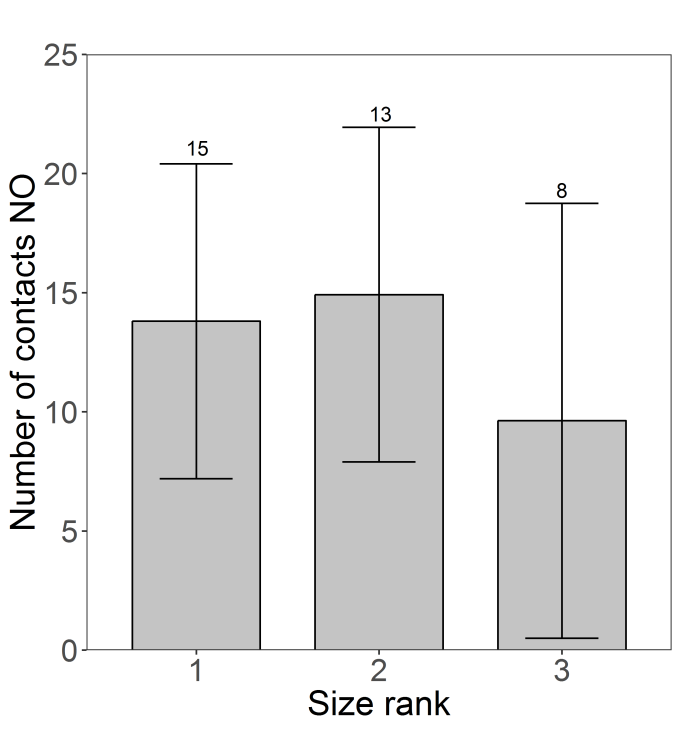


**B**

**A**

**Figure S3:** Differences in number of contacts to a novel object for pups of different size ranks (1 indicates the largest pup) over time. Panel A shows differences at the time of weaning and panel B at the time of maturation. Shown are the estimated values derived from the mixed models on the behavioural traits ± confidence intervals (CI). Samples sizes are given above the CI.


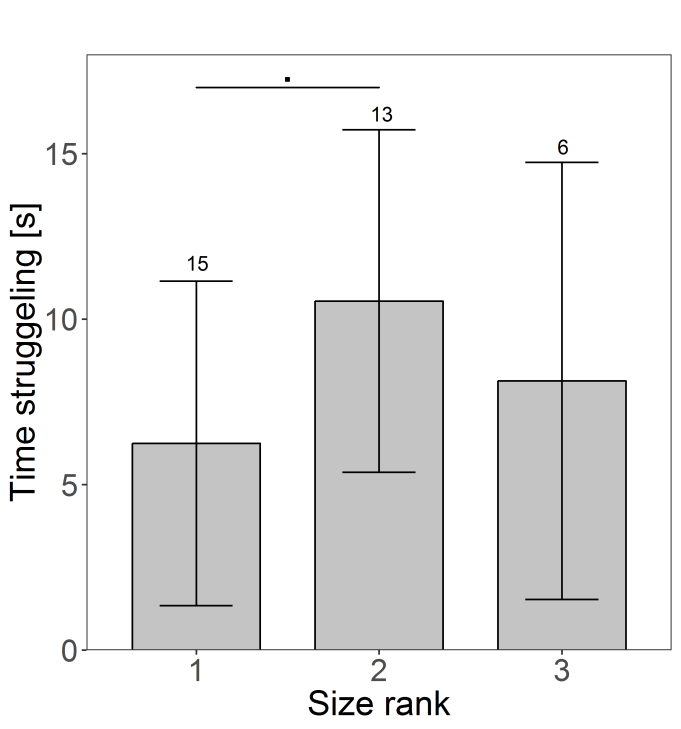

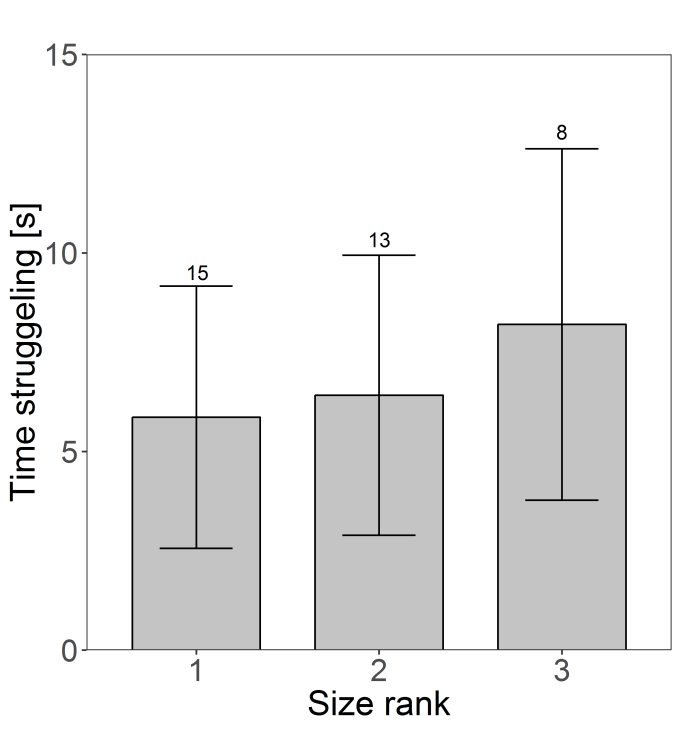


**B**

**A**

**Figure S4:** Differences in struggle docility for pups of different size ranks (1 indicates the largest pup) over time. Panel A shows differences at the time of weaning and panel B at the time of maturation. Shown are the estimated values derived from the mixed models on the behavioural traits ± confidence intervals (CI). Samples sizes are given above the CI.
